# Efficacy of a 6-Week Home-Based Online Supervised Exercise Program Conducted During COVID-19 in Patients With Post Percutaneous Coronary Intervention: A Single-Blind Randomized Controlled Trial

**DOI:** 10.3389/fcvm.2022.853376

**Published:** 2022-04-07

**Authors:** Jiajia Li, Bo Liu, Zheng Wang, Doa El-Ansary, Roger Adams, Jia Han, Shu Meng

**Affiliations:** ^1^Department of Sport Rehabilitation, School of Kinesiology, Shanghai University of Sport, Shanghai, China; ^2^Department of Cardiology, Xinhua Hospital Affiliated to Shanghai Jiaotong University School of Medicine, Shanghai, China; ^3^Department of Health Professions, Faculty of Art, Health and Design, Swinburne University of Technology, Melbourne, VIC, Australia; ^4^Research Institute for Sport and Exercise, University of Canberra, Canberra, ACT, Australia; ^5^College of Rehabilitation Sciences, Shanghai University of Medicine and Health Sciences, Shanghai, China

**Keywords:** coronary artery disease, percutaneous coronary intervention, cardiac rehabilitation, home-based, education, exercise, COVID-19

## Abstract

**Objective:**

The aim of this study was to assess the efficacy of a 6-week cardiac rehabilitation (CR) program designed for patients with coronary artery disease (CAD) after percutaneous coronary intervention (PCI) that involved an online supervised exercise program that they could access during COVID-19.

**Methods:**

One hundred patients were randomly allocated into control group (CG) and supervision group (SG). CG accepted conventional health education with a home exercise program booklet delivered before discharge, SG had an additional home-based online supervised exercise program (HOSEP). Questionnaires, motor function and lipid profile were administered at baseline. Questionnaires included the Godin-Shephard Leisure-Time Physical Activity questionnaire (GSLTPAQ) and Bandura's Exercise Self-efficacy (ESE). Motor function included: 6-min walk test (6 MWT), timed up and go test (TUG), 30-s sit to stand (30-s STS), and Hand Grip Strength (HG). Lipid profile included: low-density lipoprotein (LDL), high-density lipoprotein (HDL), total cholesterol (TC) and triglycerides (TG). The questionnaires were re-administered after 2-weeks, all tests were re-evaluated after 6-weeks.

**Results:**

the questionnaire results showed that scores on GSLTPAQ and ESE were significantly improved in the SG. The changes in GSLTPAQ scores from baseline to 2- and 6-weeks in the SG were significantly higher than in the CG (2-week: 6.9 ± 13.0 for SG and 0.2 ± 10.2 for CG, *p* = 0.005; 6-week: 9.4 ± 18.1 for SG and 0.2 ± 11.8 for CG, *p* = 0.003). in terms of motor function, both the CG and SG improved TUG and 6 MWT performance, with the 6 MWT improvement being significantly greater in the SG than CG (43.7 ± 39.2 m for SG and 16.6 ± 39.1 m for CG, *p* = 0.001). Improvement in the 30-s STS was significantly greater in the SG than CG (2.4 ± 3.6 repetitions for SG and 0.4 ± 3.5 repetitions for CG, *p* = 0.007). the lipid profile level significantly improved over baseline in both SG and CG after 6-week intervention, and these changes were not statistically different between groups.

**Conclusion:**

This pilot randomized control study demonstrated that a 6-week HOSEP, when added to education delivered pre-hospital discharge for CAD patients following PCI, was beneficial with respect to exercise self-efficacy, exercise behavior, motor function and lipid profile. Supervised exercise programs delivered online in addition to education providing effective and accessible CR during COVID-19.

## Introduction

According to the *Report on Cardiovascular Health and Diseases in China: an Updated Summary of 2020*, the prevalence of cardiovascular disease in China is 330 million, of which 11 million are coronary artery disease (CAD) (1). Percutaneous coronary intervention (PCI) is a clinical intervention to dilate and maintain patency for narrowing of the coronary arteries with significant therapeutic efficacy (2).

Cardiac rehabilitation (CR) has been recommended as an effective treatment that reduces mortality and morbidity in CAD patients after PCI (3, 4), and is supported as secondary prevention for CAD (5). CR should commence as soon as possible after hospital admission, and continue into the community upon hospital discharge (3, 6). Although the majority of patients may commence CR after PCI whilst in hospital, many cannot continue when discharged due to a shortage of programs and health professionals to support CR programs in the community (7, 8).

Home-based CR with online supervision has shown its merit in overcoming barriers of time and distance, and thus has become successful in increasing CR participation rates (7). A systematic review has demonstrated that long term (over 3 months) supervised home-based CR is not significantly different from center-based CR with respect to improving exercise capacity, risk factors and psychosocial state (8). However, the effect of short-term supervised home-based CR has not been studied and its effect on both physical and psychological measures remains unclear. Therefore, the purpose of this study was to evaluate the safety and efficacy of a 6-week home-based online supervised exercise program (HOSEP) as CR for CAD patients after PCI. The results will inform CR practice by providing evidence regarding low-cost, more convenient home-based CR, especially during the COVID-19 pandemic.

## Methods

### Participants

This study was approved by the Human Subjects Committee of Xinhua Hospital Affiliated to Shanghai Jiao Tong University school of Medicine (No. XHEC-C-2020-078-1) and registered in the Chinese Clinical Registration Center (clinical trial website: http://www.chictr.org.cn/enIndex.aspx; clinical trial registry number: Chi CTR2000037435). All Participants completed informed consent before commencement of data collection. Participants included all patients with CAD after PCI in the Department of Cardiology from Xinhua Hospital during January to December 2020. Inclusion criteria were: ➀clinically diagnosed CAD with PCI; ➁Mini Mental State Examination (MMSE) ≥24 (9); ➂native language is Chinese; ➃age ≥18 years old; ➄able to evaluate and sign informed consent on time. Exclusion criteria were: ➀left ventricular ejection fraction <40% or heart function-New York Heart Association (NYHA) grade above level III (10); ➁severe cardiac arrhythmia and cardiogenic shock; ➂combined with severe hypertension, hypertrophic cardiomyopathy, moderate to severe valvular disease, acute pericarditis or myocarditis; ➃combined with any other diseases that affect activity, such as severe liver, kidney and respiratory dysfunction, nervous system diseases, musculoskeletal system diseases, visual or auditory dysfunction; ➄had or is having CR.

### Procedure

Participants were tested at the Department of Cardiology of Xinhua Hospital during January to December 2020. All participants were blind to the intervention and randomly allocated into the Supervision group (SG) or the Control group (CG).

Baseline information, including questionnaires, motor function and lipid profile, were collected on the day before discharge.

Questionnaires were administered under the supervision of a CR physical therapist, motor function was assessed by another CR physical therapist, and lipid profile tests were done in a certified medical laboratory.

All participants had conventional face-to-face health education and the home exercise program booklet was given to the patients by a CR physical therapist before discharge. The home exercise program included walking for 30–60 min, and STS exercise for 2–3 sets every day, with scoring at 4–5 on RPE 0–10, and at 60–80% of maximum heart rate (HRmax) (HRmax = 220-age) calculated for intensity (11–13) (see Supplementary Material for an example of home exercise program). The SG had additional HOSEP, where patients were added into a WeChat virtual community and reminded every day, at 8 a.m., to 10 a.m., by CR physical therapist, to complete the walking and STS exercise as per their home exercise program.

Participants were followed up as outpatients for a 6-week period. Questionnaires were administered again at 2- and 6-weeks, motor function and lipid profile were evaluated again after the 6-week intervention. All tests were done with the same estimator and place as baseline in order to minimize sources of variability.

**Figure 1:**
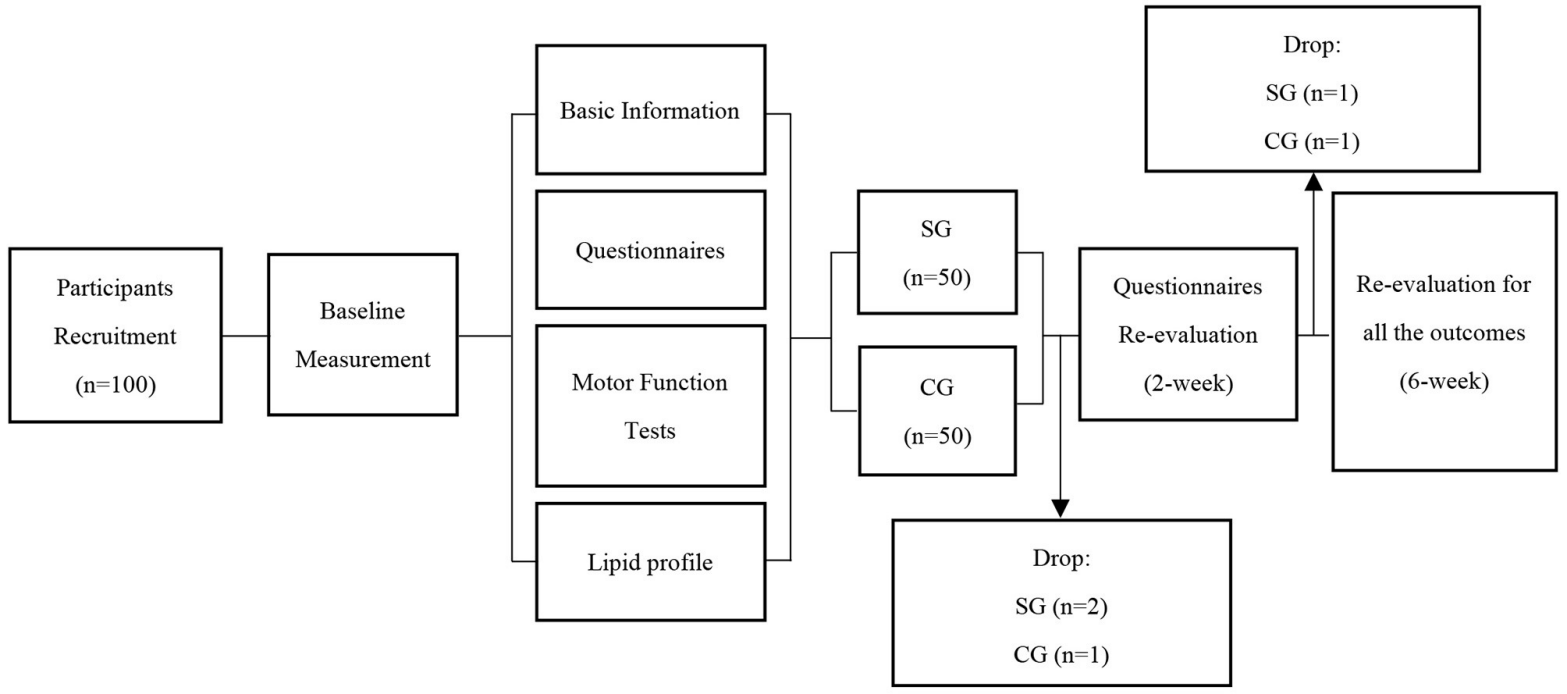
Study design and experiment flowchart. SG, Supervision group; CG, Control group.

The study design and experiment flowchart are presented in Figure 1.

### Outcome Measures

Questionnaires administered included Bandura's Exercise Self-efficacy (ESE) instrument (14) and the Godin-Shephard Leisure-Time Physical Activity Questionnaire (GSLTPAQ) (15).

Motor function tests included the 6-min Walk Test (6 MWT) (16), 30-s sit-to-stand test (30-s STS test) (17), Timed Up and Go test (TUG test) (17) and Hand Grip Strength (HG) (18). HG and TUG were assessed at first, then the 6 MWT and 30-s STS were conducted in random order. There was a 1-min interval between HG, TUG and 6 MWT, and 30 min between 6 MWT and the 30-s STS test to ensure Ratings of Perceived Exertion (RPE), heart rate and blood pressure returned to normal (18, 19). 6 MWT was conducted as per the American Thoracic Society statement (16). It was tested in a straight, unobstructed, flat corridor that was over 30 meters long. A start line was marked at one end of the corridor and the 30 m point was marked with a traffic cone as the turnaround point. For safety during the test, a chair and a source of oxygen station in the corridor were prepared. Before 6 MWT, a CR physical therapist measured patients' pulse, blood pressure, and RPE, and gave standardized instructions and encouragement. Walking distance and Post-test RPE, pulse and blood pressure were recorded.

Lipid profile included low-density lipoprotein (LDL), high-density lipoprotein (HDL), total cholesterol (TC), triglycerides (TG).

### Data Analysis

SPSS 25.0 software (IBM Corp., Armonk, NY) was used for data analysis, with intention-to-treat analysis (20). Demographic characteristics were analyzed via descriptive statistical methods. The intragroup comparison was conducted via repeated measures analysis of variance (ANOVA) and intergroup comparison of value changes by independent sample *t*-test. All results were expressed as mean ± standard deviation ($\bar{x}\pm s$) and *p* < 0.05 indicated a statistically significant difference.

## Results

### Basic Information

At the commencement of this study, 50 patients were randomly assigned for each SG and CG. At 2-week follow-up, two patients in the SG and one patient in CG dropped out for family reasons. At 6-week follow-up, one patient in each group decined to continue participation in the experiment. Therefore, 47 and 48 patients in the SG and CG, respectively completed the 6-week intervention. There were no adverse events reported during the intervention period. All patients were treated with dual antiplatelet therapy (Aspirin + Clopidogrel/Ticagrelor), β-Blocker and Stain. There were no differences between the two groups on antihypertensive (34:40, *p* = 0.17) and hypoglycemic treatments (18:15, *p* = 0.52). There were no significant intergroup differences in the demographic data at baseline (Table 1).

**Table 1:** Demographic characteristics of the patients.

	**SG**	**CG**	***p***
Age ($\bar{x}\pm s$, years)	65.3 ± 8.7	67.7 ± 7.6	0.13
Gender (male/female, cases)	35:15	39:11	0.82
BMI (kg/m^2^)	24.6 ± 3.5	24.6 ± 2.8	0.96
Risk Factor (items)	1.8 ± 1.0	2.0 ± 0.8	0.33
Occupation (full time job/retirement, cases)	12:38	8:42	0.45
Diploma (lower than high school/high school/with bachelor' s degree and above, cases)	23:16:11	19:19:12	0.71
Coronary artery lesions (single/double and above, cases)	18:32	20:30	0.68

*BMI, Body Mass Index; SG, Supervision group; CG, Control group*.

### Questionnaires

The intergroup comparison showed no statistically significant differences in the baseline questionnaire results (Table 2).

**Table 2:** Results of questionnaires ($\bar{x}\pm s$).

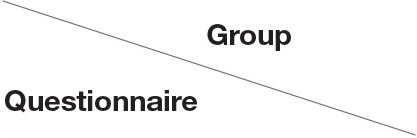	**SG**			**CG**		
	**Baseline**	**2-week**	**6-week**	**Baseline**	**2-week**	**6-week**
ESE	55.2 ± 15.3	60.8 ± 11.8	63.6 ± 9.5**	55.6 ± 12.9	59.0 ± 14.0	59.4 ± 12.3
GSLTPAQ	14.3 ± 11.8	21.2 ± 7.4**	23.7 ± 15.8**	16.8 ± 9.6	17.0 ± 5.7 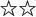	17.0 ± 8.8 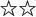

**Intragroup comparing with baseline p < 0.01.

*
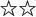
Intergroup comparison the scores change with baseline p < 0.01.*

*ESE, Bandura's Exercise Self-efficacy; GSLTPAQ, Godin-Shephard Leisure-Time Physical Activity questionnaires; SG, Supervision group; CG, Control group*.

Obtained *p* in the SG showed that, compared with baseline, the ESE scores significantly improved at 6-weeks (*p* = 0.001, 95% *CI*: 2.8–13.8), and the GSLTPAQ scores significantly improved at 2- and 6-weeks (2-week: *p* < 0.01, 95% *CI*: 2.9–10.9; 6–week: *p* < 0.01, 95% *CI*: 4.2–14.6). The intragroup comparison in the CG showed no significant differences in ESE and GSLTPAQ scores across the 3 testing points.

The intergroup comparison for the score change in GSLTPAQ showed significantly greater progress at 2- and 6-week in the SG compared with CG (2-week: 6.9 ± 13.0 vs. 0.2 ± 10.2, *p* = 0.005, 95% *CI*: 2.1–11.4. 6-week: 9.4 ± 18.1 vs. 0.2 ± 11.8, *p* = 0.003, 95% *CI*: 3.1–15.2).

### Motor Function Tests

The intergroup motor function tests comparisons showed no statistically significant differences at baseline (Table 3).

**Table 3:** Results of motor function test ($\bar{x}\pm s)$.

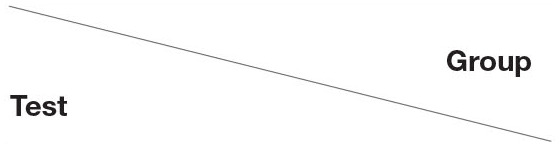		**SG**		**CG**	
		**Baseline**	**6-week**	**Baseline**	**6-week**
HG (kg)		27.2 ± 8.4	28.0 ± 8.1	26.8 ± 8.5	27.1 ± 7.9
TUG (s)		10.7 ± 3.5	9.9 ± 3.0**	10.6 ± 2.0	9.7 ± 1.9**
30-s STS (repetitions)		11.9 ± 3.5	14.3 ± 4.7**	10.9 ± 3.4	11.4 ± 3.5^I*I*^
6 MWT	B-HR (bpm)	74.9 ± 12.7	74.2 ± 10.5	76.1 ± 10.7	75.9 ± 11.3
	B-SBP (mmHg)	123.2 ± 15.2	123.6 ± 13.2	125.2 ± 19.0	127.7 ± 16.6
	B-DBP (mmHg)	71.4 ± 9.5	71.7 ± 8.3	71.5 ± 10.0	73.2 ± 10.7
	B-b-RPE	0.2 ± 0.6	0.0 ± 0.1	0.2 ± 0.6	0.1 ± 0.3
	B-l-RPE	0.1 ± 0.4	0.0 ± 0.1	0.2 ± 0.7	0.1 ± 0.3
	WD (m)	445.6 ± 36.6	489.2 ± 48.8**	436.8 ± 43.6	453.4 ± 50.7 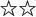
	A-HR (bpm)	81.4 ± 12.9	82.2 ± 11.5	81.0 ± 12.9	82.1 ± 13.3
	A-SBP (mmHg)	146.3 ± 16.1	147.8 ± 17.3	142.3 ± 21.9	145.8 ± 18.2
	A-DBP (mmHg)	74.4 ± 10.0	75.0 ± 11.2	71.9 ± 12.6	75.5 ± 10.5
	A-b-RPE	4.1 ± 1.2	3.5 ± 0.8**	3.8 ± 1.2	3.6 ± 0.8
	A-l-RPE	3.8 ± 1.1	3.4 ± 0.7*	3.7 ± 1.1	3.4 ± 0.7*

*Intragroup comparison with baseline p < 0.05. **Intragroup comparing with baseline p < 0.01.

*
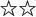
Intergroup comparison the value change with baseline p < 0.01.*

*SG, Supervision group; CG: Control group; HG, Hand Grip Strength, TUG: Timed Up and Go Test, 30-s STS: 30-second Sit to Stand; HR, Heart Rate; SBP, Systolic Blood Pressure; DBP, Diastolic Blood Pressure; B, before test; A, after test; b, breath; l, leg; RPE, Ratings of Perceived Exertion; WD, walking distance*.

Results of 30-s STS tests improved significantly only in the SG (*p* < 0.01, 95% *CI*: 1.4–3.4). In addition, the repetitions changes in the SG were significantly better than those in CG (SG: 2.4 ± 3.6 repetitions; CG: 0.4 ± 3.5 repetitions, *p* = 0.007, 95% *CI*: 0.5–3.3). The 6 MWT performance increased significantly after 6-weeks in both the SG and CG (SG: *p* < 0.01, 95% *CI*: 32.7–54.7; CG: *p* = 0.003, 95% *CI*: 5.6–27.6), and the distance changes of 6 MWT in SG were significantly better than those in CG (SG: 43.7 ± 39.2 m, CG: 16.6 ± 39.1 m, *p* = 0.001, 95% *CI*: 11.5–42.6). After 6-weeks, the RPE scores for 6 MWT decreased significantly in the SG (RPE for respiration: *p* = 0.002, 95% *CI*: 0.2–0.9; RPE for leg fatigue: *p* = 0.01, 95% *CI*: 0.1–0.7), while in the CG only lower extremity fatigue RPE decreased significantly (*p* = 0.03, 95% *CI*: 0.04–0.64). Results for the TUG after the 6-week intervention improved significantly in both groups (SG: *p* = 0.001, 95% *CI*: 0.3–1.2; CG: *p* < 0.01, 95% *CI*: 0.4–1.3). There was no significant change in HG in either group.

### Lipid Profile

The intergroup analysis showed no statistically significant differences at baseline for lipid profile (Table 4). Both LDL and HDL changed significantly compared with baseline at 6-weeks in both groups, and the value changes did not differ significantly between groups.

**Table 4:** Results of lipid profile $\left(\bar{x}\pm s\right)$.

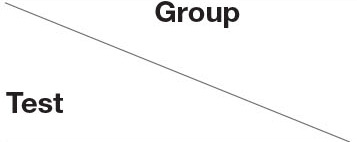	**SG**		**CG**	
	**Baseline**	**6-week**	**Baseline**	**6-week**
LDL (mmol/L)	2.2 ± 0.9	1.8 ± 0.6**	2.3 ± 0.9	1.8 ± 0.7**
HDL (mmol/L)	1.1 ± 0.3	1.2 ± 0.4**	1.1 ± 0.2	1.2 ± 0.4**
TC (mmol/L)	3.9 ± 1.0	3.7 ± 0.8	4.0 ± 1.0	4.0 ± 1.0
TG (mmol/L)	1.8 ± 1.4	1.5 ± 1.0	1.5 ± 0.8	1.3 ± 0.8

**Intragroup comparing with baseline p < 0.01.

*SG, Supervision group; CG, Control group; LDL, Low Density Lipoprotein; HDL, High Density Lipoprotein; TC, Total Cholesterol; TG, Triglycerides*.

## Discussion

This randomized controlled pilot study showed that both conventional education with a home exercise program booklet delivered before hospital discharge and an additional 6-week HOSEP were safe and effective for improving motor function and lipid profile in early Phase II community CR for CAD patients after PCI. More importantly, compared to conventional intervention, the patients who received additional HOSEP showed further improvements in their exercise self-efficacy, exercise behavior and motor function, suggesting that 6-week HOSEP, as a short-term intervention, was a feasible and valid home-based CR. These findings provide evidence regarding a low-cost, more convenient, short-term, supervised home-based CR program, especially during the COVID-19 pandemic.

In this study, WeChat (a messaging and calling app for smartphones) was used as the online supervision platform. WeChat-based CR intervention for CAD has been shown to be promising in China, with advantages such as convenience, popularity and flexibility (21). More importantly, the WeChat app has been found to be client-friendly and easy to use, even for older people (8, 22, 23). With the video call function, patients could have immediate access to medical support through their WeChat group or use one-to-one chat.

The motor function performance of both groups improved significantly. Specifically, the 6 MWT and TUG improved significantly in both groups, and the SG also improved their performance on the 30-s STS tests.

The 6 MWT is an important indicator of cardiopulmonary function in patients with CAD (16). After a 6-week intervention, both groups improved their walking distance over 450 m, indicating that their cardiopulmonary fitness may be close to normal (24). Further, the distance change in the SG was more than the minimal clinically important difference (MCID) (22), suggesting that the beneficial effect from the additional HOSEP was clinically significant. In addition, the RPE of lower extremity fatigue after 6 MWT was significantly reduced in both groups, whereas the RPE for respiratory fatigue was significantly reduced only in the SG. These findings suggest that the additional HOSEP had benefits in both musculoskeletal and respiratory systems. This is consistent with previous research, where the researchers found that aerobic exercise combined with resistance exercise improved musculoskeletal and cardiopulmonary fitness of patients with CAD (23, 25).

TUG is a test that reflects the quality of dynamic balance function (17), which involves both proprioceptive input and motor output (26). In the current study, both groups showed significant improvement in TUG but lower limb strength, i.e., motor output, only improved in the SG, suggesting that the change in observed TUG performance may be also related to proprioceptive change through exercise intervention. Future study is needed to explore the effect of CR on proprioceptive function, and how it contributes to dynamic balance control after PCI.

Lower limb muscle strength, measured by using STS, is an important contributor to CAD patients' daily living, because it has been found to be strongly associated with exercise capacity and risk of all-cause and cardiovascular mortality (27, 28). Studies by Fujita (29) found that regular STS can be used as an effective resistance exercise to improve lower extremity strength. In this study, although we prescribed for both groups to perform regular STS, only patients in the SG improved their STS performance significantly. This is likely because the HOSEP improved patients' motivation to perform the hop exercise regularly.

For the lipid profile, the blood results showed that HDL and LDL were more ideal in both groups after 6-weeks of CR. The results were comparable to other long-term HOSEP studies (30, 31), suggesting that short-term HOSEP can also benefit participants' lipid profile. Additionally, research has shown that long-term (more than 3 months intervention) HOSEP can also improve TC and TG (32). However, we did not find any significant change in TC and TG in this short-term (6 week) study. Further work is needed to determine how long the intervention needs to be to achieve effective change in TC and TG. In addition, there is a need to consider including more biochemical/physiological parameters, such as blood morphology, electrocardiogram, or respiratory fitness tests, in that this information feedback may contribute to greater patient motivation to continue physical activity.

The questionnaire results showed that the score change on GSLTPAQ in the SG was significantly higher than the CG. Scores for ESE significantly improved between baseline and 6 weeks in the SG. These findings suggest that patients in the HOSEP group may have developed a better sense of self-efficacy and exercised more regularly at home, a result which is also similar to the effects found in a long-term online exercise study (33).

## Conclusion

Both conventional education and the additional 6-week HOSEP were safe and beneficial with respect to motor function and lipid profile. HOSEP can further improve patients' exercise behavior and motor function over conventional treatment. Therefore, a 6-week HOSEP in early phase II CR should be considered as an effective intervention as it can be conducted at home, especially during the COVID-19 pandemic, and it provides safe access to all patients following PCI in the community, thereby reducing demand on services.

## Data Availability Statement

The original contributions presented in the study are included in the article/Supplementary Material, further inquiries can be directed to the corresponding authors.

## Ethics Statement

The studies involving human participants were reviewed and approved by Human Subjects Committee of Xinhua Hospital Affiliated to Shanghai Jiao Tong University School of Medicine. The patients/participants provided their written informed consent to participate in this study.

## Author Contributions

JL, ZW, BL, JH, and SM designed the study and had primary responsibility for this work. JL, ZW, and BL responsible for the execution of the trial and data collection. JL and ZW analyzed the data and discussed with JH and SM. JL wrote the first draft, which was improved by SM and JH. DE-A and RA critically reviewed and improved the manuscript. All authors read and approved the final manuscript.

## Funding

This study was supported by the 2021 Curriculum Construction Project of Xinhua Hospital affiliated to Shanghai Jiaotong University School of Medicine.

## Conflict of Interest

The authors declare that the research was conducted in the absence of any commercial or financial relationships that could be construed as a potential conflict of interest.

## Publisher's Note

All claims expressed in this article are solely those of the authors and do not necessarily represent those of their affiliated organizations, or those of the publisher, the editors and the reviewers. Any product that may be evaluated in this article, or claim that may be made by its manufacturer, is not guaranteed or endorsed by the publisher.
