# Supplementary material for: Efficacy of a 6-Week Home-Based Online Supervised Exercise Program Conducted During COVID-19 in Patients With Post Percutaneous Coronary Intervention: A Single-Blind Randomized Controlled Trial
Source: Front Cardiovasc Med. 2022 Apr 7;9:853376. doi: 10.3389/fcvm.2022.853376 (PMC9021490; doi:10.3389/fcvm.2022.853376)
Supplement: Supplementary file 2 [file Table_2.DOCX]

| An Example of the Home Exercise Program | | |
| --- | --- | --- |
| Age | | 60 years |
| 6MWT result | | 480 m |
| 30-s STS testing result | | 18 repetitions |
| Home exercise program | Walk | **Duration:** 30 min  **Goal:** 1920m (80%*5*6MWT result=1920m)  Frequency: every day  **Intensity:** HR=96-128 (60%-80% HRmax=220-60) or RPE=4-5 |
|  | STS | **Sets:** 3  **Repetitions per set:** 30-36 (80%-100%*2*30s-STS)  **Intensity:** HR=96-128 (60%-80%HRmax=220-60) or RPE=4-5  **Rest between sets:** 30-60 s  **Frequency:** every day |
| Note: 6MWT: Six-minute Walk Test; 30-s STS: 30-second Sit to Stand | | |
