# Supplementary material for: Efficacy of a 6-Week Home-Based Online Supervised Exercise Program Conducted During COVID-19 in Patients With Post Percutaneous Coronary Intervention: A Single-Blind Randomized Controlled Trial
Source: Front Cardiovasc Med. 2022 Apr 7;9:853376. doi: 10.3389/fcvm.2022.853376 (PMC9021490; doi:10.3389/fcvm.2022.853376)
Supplement: Supplementary file 3 [file Data_Sheet_1.ZIP › supplementary files 3/English version-Bandura's exercise self-efficacy scale.pdf]

A survey about confidence and exercise

We would like to know how confident you are that you can exercise regularly under different circumstances. For each statement, please put a checkmark in the box that best matches your level of confidence.

How confident are you that you can exercise most days of the week:

	Not confident at all<br>1	2	Somewhat confident<br>3	4	Very confident<br>5
When I feel tired	☐	☐	☐	☐	☐
When I am under pressure at work	☐	☐	☐	☐	☐
During bad weather	☐	☐	☐	☐	☐
After recovering from an illness or injury that caused me to stop exercising	☐	☐	☐	☐	☐
When I am having personal problems	☐	☐	☐	☐	☐
When I am having family problems	☐	☐	☐	☐	☐
When I feel depressed	☐	☐	☐	☐	☐
When I feel anxious	☐	☐	☐	☐	☐
When exercise is uncomfortable	☐	☐	☐	☐	☐
During a holiday	☐	☐	☐	☐	☐
After a holiday	☐	☐	☐	☐	☐
When I have a lot of work to do at home	☐	☐	☐	☐	☐
When I have visitors	☐	☐	☐	☐	☐
When I am busy	☐	☐	☐	☐	☐
When I have other interesting things to do	☐	☐	☐	☐	☐
Without support from my family or friends	☐	☐	☐	☐	☐
Even if I don’t reach my exercise goals	☐	☐	☐	☐	☐

Thank you for taking part in our research.

Exercise Self-Efficacy Scale
