# Supplementary material for: Efficacy of a 6-Week Home-Based Online Supervised Exercise Program Conducted During COVID-19 in Patients With Post Percutaneous Coronary Intervention: A Single-Blind Randomized Controlled Trial
Source: Front Cardiovasc Med. 2022 Apr 7;9:853376. doi: 10.3389/fcvm.2022.853376 (PMC9021490; doi:10.3389/fcvm.2022.853376)
Supplement: Supplementary file 3 [file Data_Sheet_1.ZIP › supplementary files 3/Chinese version-Godin Leisure-Time Exercise Questionnaire.pdf]

体力活动：戈丁问卷

戈丁业余时间运动问卷

说明：这是戈丁业余时间运动问卷的节选，需要独立完成四项简短的关于业余时间运动习惯的问题。

戈丁业余时间运动问卷

1. 在典型的 7 天中（1 周），您在空闲时间做以下强度的运动超过 15 分钟的次数（将数字填写在横线上）。

每周的次数

A）剧烈运动 (心跳很快)
(例如：跑步，足球，篮球，长距离骑自行车) __________

B）中等程度的运动（未到筋疲力尽的程度）
(例如：快走，慢跑，轻松的骑自行车) __________

C）低等程度的运动（稍微用力）
(例如散步，瑜伽，钓鱼，家务劳动，遛狗，带小孩) __________

2. 在 7 天（1 周）中，您在业余时间内能有规律的进行几次长时间运动至出汗的项目（心跳很快）？

经常	有时	从不/偶尔
1.☐	2.☐	3.☐

计算：在第一个问题中，每周剧烈、中度和轻度活动的频率分别乘以 9、5、3。每周业余运动总量，即为各部分乘积的总和。计算公式如下：

每周业余运动评分=(9×剧烈)+(5×中度)+(3×轻度)；

第二个问题用于计算每周完成“可长时间运动到流汗”项目的频率。

例子：剧烈=3 次/周
中度=6 次/周
轻度=14 次/周
总的业余时间运动得分=（9*3）+（5*6）+（3*14）=27+30+42=99
