# Supplementary material for: Efficacy of a 6-Week Home-Based Online Supervised Exercise Program Conducted During COVID-19 in Patients With Post Percutaneous Coronary Intervention: A Single-Blind Randomized Controlled Trial
Source: Front Cardiovasc Med. 2022 Apr 7;9:853376. doi: 10.3389/fcvm.2022.853376 (PMC9021490; doi:10.3389/fcvm.2022.853376)
Supplement: Supplementary file 3 [file Data_Sheet_1.ZIP › supplementary files 3/English version-Godin Leisure-Time Exercise Questionnaire.pdf]

Godin Leisure-Time Exercise Questionnaire

INSTRUCTIONS

In this excerpt from the Godin Leisure-Time Exercise Questionnaire, the individual is asked to complete a self-explanatory, brief four-item query of usual leisure-time exercise habits.

CALCULATIONS

For the first question, weekly frequencies of strenuous, moderate, and light activities are multiplied by nine, five, and three, respectively. Total weekly leisure activity is calculated in arbitrary units by summing the products of the separate components, as shown in the following formula:

Weekly leisure activity score = (9 × Strenuous) + (5 × Moderate) + (3 × Light)

The second question is used to calculate the frequency of weekly leisure-time activities pursued “long enough to work up a sweat“ (see questionnaire).

EXAMPLE

Strenuous = 3 times/wk

Moderate = 6 times/wk

Light = 14 times/wk

Total leisure activity score = (9 × 3) + (5 × 6) + (3 × 14) = 27 + 30 + 42 = 99

Godin Leisure-Time Exercise Questionnaire

1. During a typical **7-Day period** (a week), how many times on the average do you do the following kinds of exercise for **more than 15 minutes** during your free time (write on each line the appropriate number).

Times Per Week

a) **STRENUOUS EXERCISE**
(HEART BEATS RAPIDLY) __________
(e.g., running, jogging, hockey, football, soccer, squash, basketball, cross country skiing, judo, roller skating, vigorous swimming, vigorous long distance bicycling)

b) MODERATE EXERCISE
(NOT EXHAUSTING) ____________
(e.g., fast walking, baseball, tennis, easy bicycling,
volleyball, badminton, easy swimming, alpine skiing,
popular and folk dancing)

c) MILD EXERCISE
(MINIMAL EFFORT) ____________
(e.g., yoga, archery, fishing from river bank, bowling,
horseshoes, golf, snow-mobiling, easy walking)

2. During a typical **7-Day period** (a week), in your leisure time, how often do you engage in any regular activity **long enough to work up a sweat** (heart beats rapidly)?

OFTEN	SOMETIMES	NEVER/RARELY
1. ☐	2. ☐	3. ☐
