# Supplementary material for: Efficacy of a 6-Week Home-Based Online Supervised Exercise Program Conducted During COVID-19 in Patients With Post Percutaneous Coronary Intervention: A Single-Blind Randomized Controlled Trial
Source: Front Cardiovasc Med. 2022 Apr 7;9:853376. doi: 10.3389/fcvm.2022.853376 (PMC9021490; doi:10.3389/fcvm.2022.853376)
Supplement: Supplementary file 3 [file Data_Sheet_1.ZIP › supplementary files 3/Chinese version-Bandura's exercise self-efficacy scale.pdf]

# 自我效能：班杜拉运动自我效能量表（改良版）

## 关于自信心和运动的调查

我们想要了解您在不同环境下，进行规律运动的自信程度。对于以下每一个陈述，请在最符合您自信水平下的方框中打勾。

**在一周内大多数时间的运动中您的自信程度如何?**

| | 一点也不自信 1 | 不太自信 2 | 有点自信 3 | 比较自信 4 | 非常自信 5 |
|---|---|---|---|---|---|
| 当我感觉疲劳时 | ☐ | ☐ | ☐ | ☐ | ☐ |
| 当我工作或生活有压力时 | ☐ | ☐ | ☐ | ☐ | ☐ |
| 当天气糟糕时 | ☐ | ☐ | ☐ | ☐ | ☐ |
| 当导致我无法运动的疾病或损伤痊愈后 | ☐ | ☐ | ☐ | ☐ | ☐ |
| 当遇到个人问题时 | ☐ | ☐ | ☐ | ☐ | ☐ |
| 当遇到家庭问题时 | ☐ | ☐ | ☐ | ☐ | ☐ |
| 当感到沮丧时 | ☐ | ☐ | ☐ | ☐ | ☐ |
| 当感到焦虑时 | ☐ | ☐ | ☐ | ☐ | ☐ |
| 在运动中感觉到不适时 | ☐ | ☐ | ☐ | ☐ | ☐ |
| 在假期期间 | ☐ | ☐ | ☐ | ☐ | ☐ |
| 在假期后 | ☐ | ☐ | ☐ | ☐ | ☐ |
| 当我在家有很多事情要做时 | ☐ | ☐ | ☐ | ☐ | ☐ |
| 当有客人时 | ☐ | ☐ | ☐ | ☐ | ☐ |
| 当忙碌时 | ☐ | ☐ | ☐ | ☐ | ☐ |
| 当有其他感兴趣的事要做时 | ☐ | ☐ | ☐ | ☐ | ☐ |
| 当没有家人或朋友的支持时 | ☐ | ☐ | ☐ | ☐ | ☐ |
| 当没有达到运动目标时 | ☐ | ☐ | ☐ | ☐ | ☐ |

**感谢您参与运动自我效能的调查！**
